# Evolution, Composition and Regulation of Supernumerary B Chromosomes

**DOI:** 10.3390/genes10020161

**Published:** 2019-02-20

**Authors:** Andreas Houben, Neil Jones, Cesar Martins, Vladimir Trifonov

**Affiliations:** 1 Leibniz Institute of Plant Genetics and Crop Plant Research (IPK), Corrensstrasse 3, 06466 Gatersleben, Germany; 2 Aberystwyth University, Institute of Biological, Environmental and Rural Sciences (IBERS), Edward Llwyd Building, Penglais Campus, Aberystwyth SY23 3DA, UK; rnj@aber.ac.uk; 3 Institute of Bioscience at Botucatu, São Paulo State University—UNESP, Botucatu, SP 18618, Brazil; cesar.martins@unesp.br; 4 Laboratory of Comparative Genomics, Department of the Diversity and Evolution of Genomes, Institute of Molecular and Cellular Biology SB RAS, Novosibirsk 630090, Russia; vlad@mcb.nsc.ru

Supernumerary B chromosomes (Bs) are dispensable genetic elements found in thousands of species of plants and animals, and some fungi. Since their discovery more than a century ago, they have been a source of puzzlement, as they only occur in some members of a population and are absent from others. When they do occur, they are often harmful, and in the absence of “selfishness”, based on mechanisms of mitotic and meiotic drive, there appears to be no obvious reason for their existence. Cytogeneticists have long wrestled with questions about the biological existence of these enigmatic elements, including their lack of any adaptive properties, apparent absence of functional genes, their origin, sequence organization, and co-evolution as nuclear parasites. Emerging new technologies are now enabling researchers to step up a gear, to look enthusiastically beyond the previous limits of the horizon, and to uncover the secrets of these “silent” chromosomes.

This volume, “Evolution, Composition and Regulation of Supernumerary B Chromosomes”, consists of a series of new reviews and original research articles, and provides a comprehensive guide to theoretical advancements in the field of B chromosome research in both animal and plant systems. Beside “classical” B chromosomes, supernumerary A chromosomal segments and germ-line limited (termed L or “limited”) chromosomes will be addressed. The topics include investigations into their DNA composition, transcriptional activity, and effects on the host transcriptome profile, drive, origin, and evolution.

Among animals, B chromosomes have been largely investigated in insects, fish, and mammals. Based on the study of nine grasshopper species from Europe, Asia, and Africa, Jetybayev et al. [1] state that the origin and evolution of Bs depend on particularities, such as the hotspots of chromosome rearrangements, the mobility of genomic elements, and the tendency of specific DNA fragments towards amplification. Milani et al. [2] use a combination of cytogenetics and bioinformatics to investigate Bs in three other grasshopper species from North and South America. Their data show the recurrent involvement of small A chromosomes, that are poor in genes and enriched in repetitive DNA, in the B chromosome origin. Hanlon and Hawley [3] present a review on B studies in *Drosophila,* and highlight *D. melanogaster* as a versatile model to advance our understanding of the complex B chromosome biology. 

Another interesting animal group for B studies is represented by fish species. Komissarov et al. [4] investigate Bs of the Asian Seabass (*Lates calcarifer*), and give support to the view that Bs contribute to the variations in the genome among individuals and populations of the species. Among fish, cichlids have appeared as an interesting model for B studies. Coan and Martins [5] demonstrate that expanded B transposable elements are under functional control and are not highly transcribed. Clark et al., [6] on the basis of both Illumina and PacBio sequence data of seven cichlid species, have accessed a “core” B chromosome dataset of genes and fragmented genes.

Among amniotes, Bs have been much better studied in mammals than in reptiles, birds, and amphibians. Although there are many reports on B chromosomes in amphibian species, only few studies have been done in lizards and no Bs were reported in avian genomes. This may reflect the fact that most amphibian and reptile genomes are poorly studied, and birds might lack Bs due to their characteristic genome evolution and karyotype structure. However, in songbirds, the Germline-Restricted Chromosome (GRC) has been reported [7,8]. 

Two review papers on B chromosomes in mammals—by Rubtsov and Borisov [9] and by Vujosevic et al. [10] —are devoted to B chromosome origin, content, activity, and evolution in mammals. These two articles complement each other, as they address Bs from different perspectives. Both reviews summarize recent data obtained from different mammalian species, and make several important conclusions: They suggest a considerable heterogeneity of mammalian Bs based on their origin and subsequent evolution. Bs found in modern species seem to be different in their origin and have undergone different evolutionary trajectories, although they might have been shaped by similar evolutionary mechanisms. Rubtsov and Borisov [9] suggest several models of B chromosome origin, while the article by Vujosevic et al. [10] provides an updated list of mammalian species with Bs, and gives a detail description of research experiments accomplished on these species.

The research article by Makunin et al. [11] provides novel data on B chromosome content and evolution in the red fox (the first mammalian species with Bs, whose genome has recently been sequenced and assembled (Kukekova et al.) [12], and in the raccoon dog, the carnivore species, where B-specific coding genes were discovered almost 13 years ago. Using new generation sequencing, the authors argue that the origin of B chromosome in these species is independent. Through the analysis of B content in different mammals they conclude a frequent and independent re-use of the same genomic regions in B chromosome formation. They suggest that such a re-use may be connected with gene functions.

Borisov and Zhigarev [13] consider another mammalian species—*Apodemus peninsulae*—a good model for B chromosome studies. They summarize their data, collected over 40 years in different geographical regions of species distribution, and conclude that the variability of B chromosome systems results from stochastic processes in populations. 

Another important set of contributions come from plants. H. Su et al. [14] discuss the maize B alongside the latest progress of centromere activities, including centromere mis-division, inactivation, reactivation and de novo centromere formation. Drive is one of the most important B chromosome features. R. N. Jones [15] summarizes the mechanisms of drive, which enable B chromosomes to enhance their transmission rates by various processes of non-Mendelian inheritance in plants and animals. A. Marques et al. [16] demonstrate how new genomic approaches have shed light on the origin and accumulation of different classes of repetitive sequences in the process of B chromosome formation and evolution. M. Dhar et al. [17] summarize the characterization of a novel B which was discovered in *Plantago lagopus*. This B was found to be composed of mainly 5S rDNA-derived sequences and various types of repetitive elements. The transmission of the *Plantago* B through the female sex track does not follow Mendelian principles. 

Supernumerary chromosomal segments represent additional chromosomal material that, unlike B chromosomes, is attached to the standard A chromosome complement. Using the *Prospero autumnale* complex (Hyacinthaceae) as a model, T-S. Jang et al. [18] decipher the possible origin of supernumerary chromosomal segments as by-products of the extensive genome restructuring within a putative ancestral *P. autumnale* genome, predating the complex diversification at the diploid level and perhaps linked to B chromosome evolution.

The germ-line limited (termed L or “limited”) supernumerary chromosomes of the dipteran *Sciara coprophila* was revised by Singh and Belyakin, [19] adding knowledge to the imprinting phenomenon of such extra elements. 

We believe that this volume will be an important resource for a wide variety of audiences, including junior graduate students and established investigators who are interested in chromosome biology and genome evolution.
